# Refractory cor pulmonale under extracorporeal membrane oxygenation for acute respiratory distress syndrome: the role of conversion to veno-pulmonary arterial assist—a case series

**DOI:** 10.3389/fmed.2024.1348077

**Published:** 2024-04-25

**Authors:** François Bagate, Paul Masi, Madjid Boukantar, Costin Radu, Gabriel Saiydoun, Antonio Fiore, Paul-Matthieu Chiaroni, Emmanuel Teiger, Thierry Folliguet, Romain Gallet, Armand Mekontso Dessap

**Affiliations:** ^1^AP-HP, Hôpitaux Universitaires Henri Mondor, DHU A-TVB, Service de Médecine Intensive Réanimation, Créteil, France; ^2^Université Paris Est Créteil, Faculté de Médecine, Groupe de recherche clinique CARMAS, Créteil, France; ^3^APHP, Hôpitaux Universitaires Henri Mondor, Service de Cardiologie, Créteil, France; ^4^APHP, Hôpitaux Universitaires Henri Mondor, Département de Chirurgie Cardiaque, Créteil, France; ^5^U955-IMRB, Equipe 03, Inserm, Univ Paris Est Créteil (UPEC), Ecole Nationale Vétérinaire d’Alfort (EnVA), Maisons-Alfort, France; ^6^INSERM U955, Institut Mondor de Recherche Biomédicale, Créteil, France

**Keywords:** acute respiratory distress syndrome, acute cor pulmonale, veno-pulmonary arterial extracorporeal membrane oxygenation, veno-pulmonary arterial assist, right ventricle assist device, case series

## Abstract

**Introduction:**

Pulmonary vascular dysfunction during severe acute respiratory distress syndrome (ARDS) may lead to right ventricle (RV) dysfunction and acute cor pulmonale (ACP). The occurrence/persistence of ACP despite conventional extracorporeal membrane oxygenation (ECMO) is a challenging situation. We explored the usefulness of a specific dual-lumen cannula that bypasses the RV, and on which a veno-pulmonary arterial assist (V-P ECMO) was mounted, in ARDS patients.

**Methods:**

We report a case-series of ARDS patients put on conventional veno-arterial or veno-venous ECMO and presented refractory ACP as an indication for a reconfiguration to V-P ECMO using the ProtekDuo cannula. The primary endpoint was the mitigation of RV and pulmonary vascular dysfunction as assessed by the change in end-diastolic RV/left ventricle (LV) surface ratio.

**Results:**

Six patients had their conventional ECMO reconfigured to V-P ECMO to treat refractory ACP. There was a decrease in end-diastolic RV/LV surface ratio, as well as end-systolic LV eccentricity index, and lactatemia immediately after V-P ECMO initiation. The resolution of refractory ACP was immediately achieved in four of our six (66%) patients. The V-P ECMO was weaned after a median of 26 [8–93] days after implantation. All but one patient were discharged home. We detected one case of severe hemolysis with V-P ECMO and two suspected cases of right-sided infective endocarditis.

**Conclusion:**

V-P ECMO is useful to mitigate RV overload and to improve hemodynamics in case of refractory ACP despite conventional ECMO.

## Introduction

Right ventricular (RV) dysfunction secondary to increased pulmonary vascular resistance is a common manifestation of acute respiratory distress syndrome (ARDS) (1–3). The underlying mechanisms (4) of RV dysfunction include vascular occlusion (driven by endothelial dysfunction, micro-thrombi, and vascular remodeling), constriction (driven by hypoxemia, hypercapnia (5–7) and inflammatory mediators, as well as lung fibrosis), and compression (by positive pressure mechanical ventilation) (8). Severe pulmonary vascular dysfunction often leads to acute cor pulmonale (ACP) and RV dysfunction, which are associated with poor outcomes in ARDS (1, 2, 9, 10). In case refractory hypoxemia and/or hypercapnia develop despite the use of optimized mechanical ventilation and prone positioning, the implantation of veno-venous extracorporeal membrane oxygenation (V-V ECMO) is considered (11, 12). The latter reduces RV afterload by reversing hypoxemia, hypercapnia, and acidemia (13, 14).

However, sometimes ACP persists despite the use of V-V ECMO and other measures to protect the RV (nitric oxide, ultra-protective mechanical ventilation, prone position) (15), which substantially compromise patient prognosis due to the scarcity of alternative solutions (16). One of those solutions consists in converting V-V ECMO to veno-arterial (V-A) or veno-arterial and venous (V-A-V) ECMO (17). A recent alternative, with venous single-access and probably safer cannulation, is to reconfigure the standard ECMO to veno-pulmonary arterial ECMO (V-P ECMO) to bypass the RV (18, 19). With this technique, a single, dual-lumen cannula (ProtekDuo, LivaNova, London, United Kingdom), connected to an oxygenator, is inserted percutaneously through the right internal jugular vein and passes the RV to draw desaturated blood from the right atrium and return reoxygenated and decarboxylated blood into the pulmonary artery.

V-P ECMO may therefore improve RV function and outcomes of patients exhibiting refractory ACP despite conventional ECMO. The purpose of this case series was to describe the feasibility of reconfiguring conventional ECMO to percutaneous V-P ECMO in patients with refractory ACP.

## Method

### Study design and patients

This case series enrolled consecutive patients from August 2021 to October 2022, in the medical intensive care unit (ICU) of a tertiary university hospital hosting a mobile circulatory assistance unit. Patients put on conventional V-V or V-A ECMO were included if their ECMO were reconfigured to V-P ECMO to treat refractory ACP. The criteria for V-V ECMO implantation were those used in EOLIA trial (11). Refractory ACP was defined by the combination of the following criteria: (i) persistence of severe RV dilatation (end-diastolic RV/LV area ratio > 1) with interventricular septal dyskinesia on echocardiography; (ii) hemodynamic instability (defined as the need for vasopressor support and/or arterial lactatemia of >2 mmol/L); (iii) using RV protective measures combining V-V ECMO to correct hypoxemia and hypercapnia, ultraprotective ventilation (with target plateau pressure ≤ 24 cmH_2_O, PEEP ≤12 cmH_2_O, and respiratory rate ≤ 20 breaths/min, whenever possible), and either prone position or nitric oxide, if the former was deemed impossible.

In accordance with the ethical standards of our hospital’s institutional review board and the French law, all patients or their close relatives were informed that their personal data were collected in this research and that they could decline inclusion. The study protocol was approved by Henri Mondor University Hospital ethics committee (registration code no. 1,778,041). This case series was written according to the CARE guideline (https://www.care-statement.org, CAse REport guidelines (CARE) checklist provided in Supplementary material 1).

### Data collection

We collected data on baseline characteristics, ventilation, hemodynamics and ECMO management before and after the ECMO reconfiguration, as well as clinical outcomes. The vasoactive inotropic score (VIS) was defined as: dobutamine dose (μg/kg/min) + 100*epinephrine dose (μg/kg/min) + 100*norepinephrine base dose (μg/kg/min) (20). In addition, and whenever available, the following echocardiographic parameters were collected within approximately two hours before and after implementing the V-P ECMO: left ventricle ejection fraction, left ventricular global longitudinal strain, velocity–time integral of left ventricular outflow tract, cardiac index, end-systolic LV eccentricity index, systolic pulmonary arterial pressure (sPAP), fractional area change of right ventricle (FAC-RV), tricuspid annular diameter, tricuspid annular plane systolic excursion (TAPSE), peak systolic tricuspid annulus velocity (obtained using pulsed tissue Doppler), right ventricular global longitudinal strain, and RV free wall longitudinal strain. FAC-RV/sPAP ratio and TAPSE/sPAP were chosen as surrogates of RV-pulmonary artery coupling (normal values of FAC-RV/sPAP are >0.94) (21).

### Procedures

**Figure 1:**
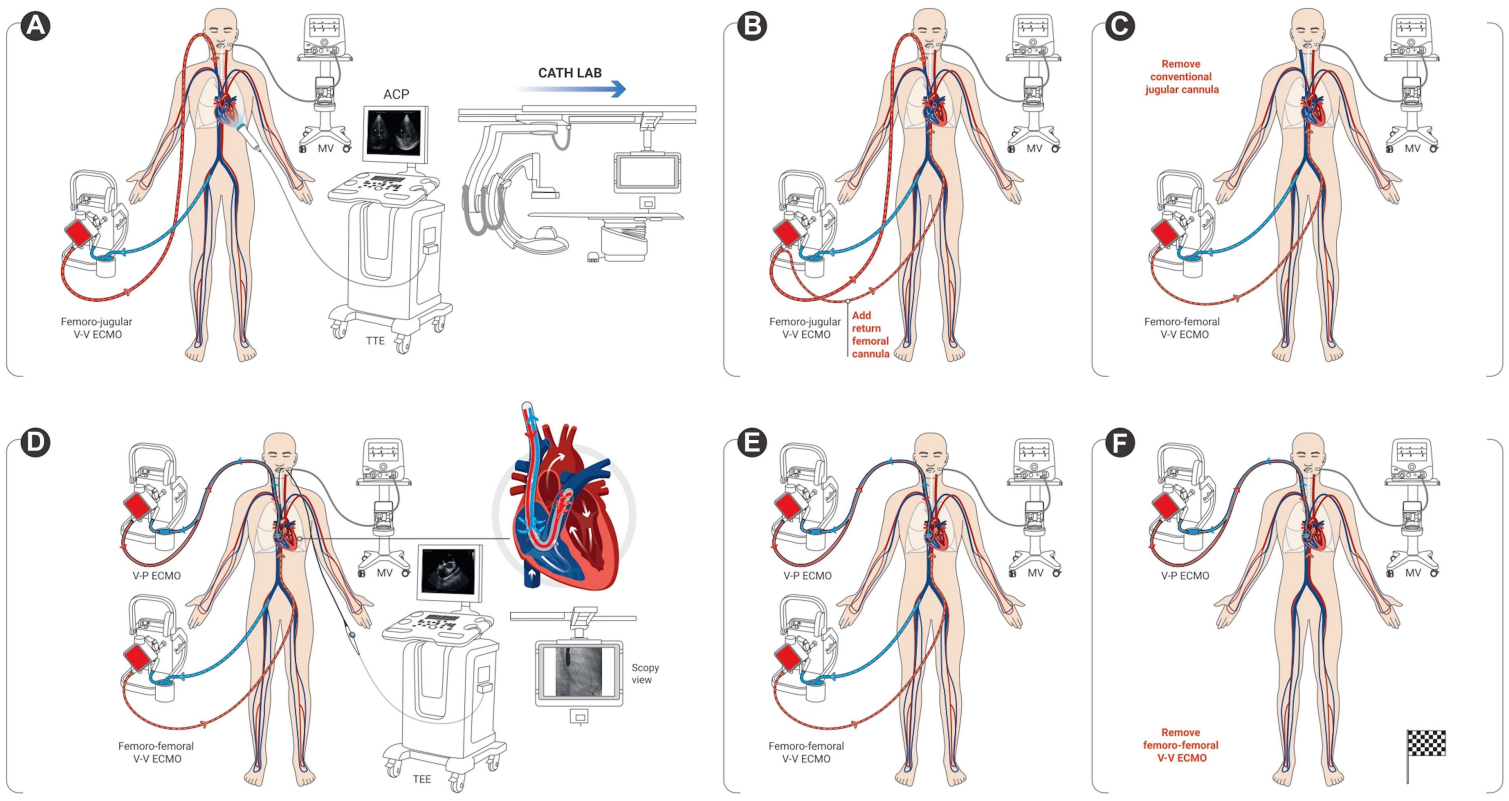
Conversion process from conventional femoro-jugular veno-venous extracorporeal membrane oxygenation (V-V ECMO) to veno-pulmonary ECMO. **(A)** patient with femoro-jugular veno-venous extracorporeal membrane oxygenation (V-V ECMO) and refractory acute cor pulmonale (ACP) seen on transthoracic echocardiography (TTE), is transferred to the catheterization laboratory (cath lab); **(B)** additional return femoral cannula; **(C)** return jugular cannula is removed for transitory femoro-femoral V-V ECMO configuration; **(D)** percutaneous insertion of the single, dual-lumen cannula (ProtekDuo) through the right jugular vein, and advanced into the main pulmonary artery under dual fluoroscopic and trans-esophageal (TEE) guidance; **(E)** transitory dual ECMO configuration; **(F)** veno-pulmonary extracorporeal membrane oxygenation (V-P ECMO) is set on after removal of the femoro-femoral V-V ECMO.

**Figure 2:**
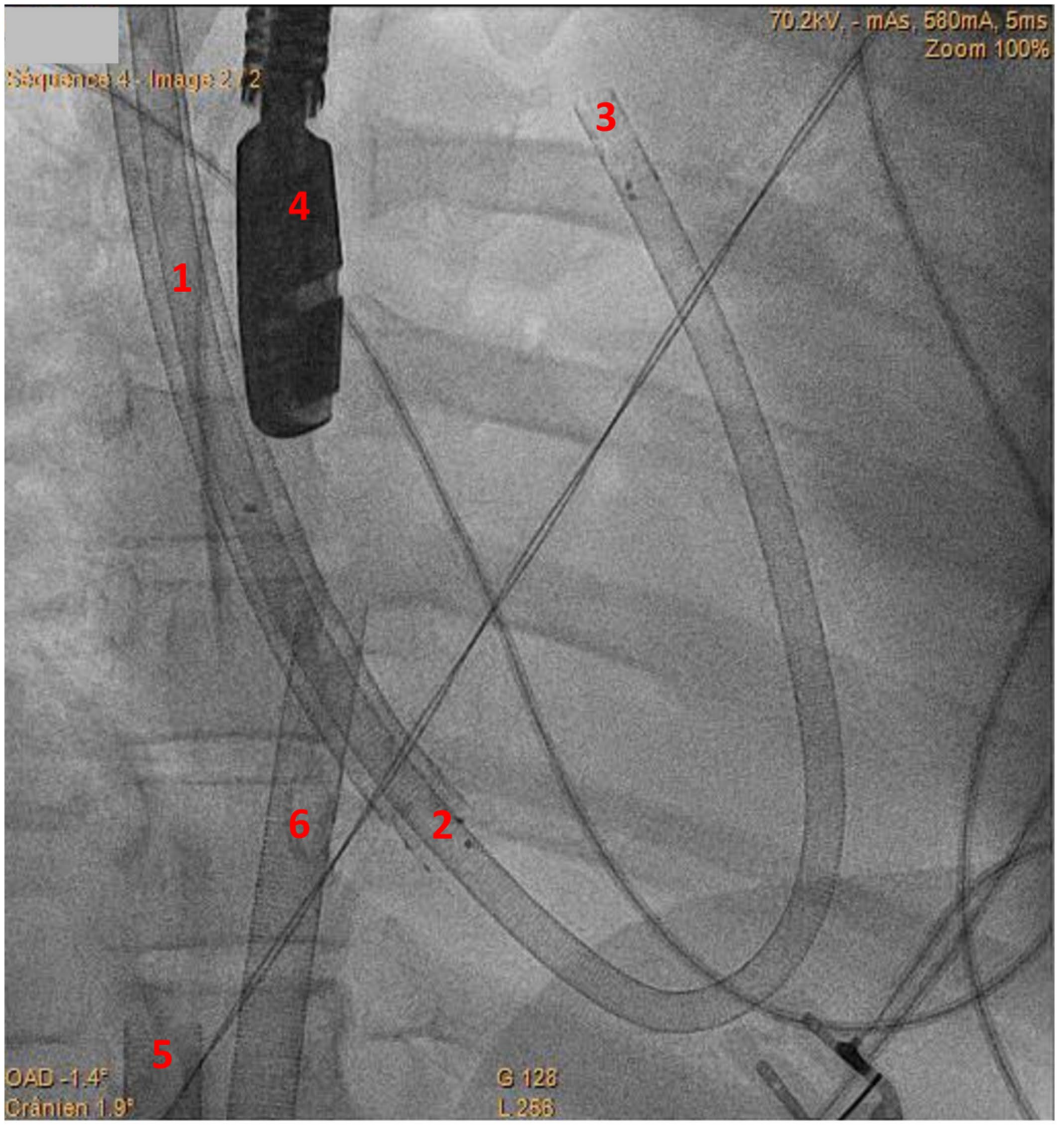
Fluoroscopy image of ProtekDuo cannula implantation. 1: ProtekDuo cannula in superior vena cava; 2: Inflow lumen of ProtekDuo cannula in place in right atrium; 3: tip of the ProtekDuo cannula (i.e., outflow lumen) in place in the main pulmonary artery; 4: tip of trans-esophageal echocardiography probe; 5: venous drainage cannula of femoro-femoral venovenous ECMO; 6: return cannula of femoro-femoral venovenous ECMO.

All technical procedures were performed on patients intubated and sedated, in the cardiac catheterization laboratory under dual fluoroscopic and trans-esophageal echocardiography (TEE) guidance. Four patients needed a transitory femoro-femoral cannulation in order to access the right jugular vein upon reconfiguration (Figure 1). The right jugular vein was punctured under ultrasound guidance using the Seldinger’s technique. After preparation with adequate dilatators, the right jugular vein was then cannulated using a stiff wire (Amplatz Super Stiff^®^, Boston Scientific, Marlborough, Massachusetts, United States) under fluoroscopic guidance. The 29 Fr ProtekDuo cannula was inserted percutaneously through the right jugular vein and threaded into the main pulmonary artery under dual fluoroscopic and TEE guidance (Figure 2 and Supplementary material 2—video 1).

### Statistical analyses

Statistical analyses were performed with the GraphPad Prism software (version 5; GraphPad Software Inc., La Jolla, CA, United States). The primary endpoint of the study was the change in end-diastolic RV/LV surface ratio measured on four-chamber apical view of transthoracic echocardiography (22). Data were presented as medians with interquartile ranges or numbers with percentages, as appropriate. Given the essentially descriptive nature of our study, no statistical comparisons were made.

## Results

### Study population

During the study period, 25 patients were put on conventional ECMO to treat their refractory ARDS (22 V-V ECMO and 3 V-A-V ECMO). Three additional patients were primarily assisted by V-A ECMO for refractory ARDS with severe ACP. Overall, we included six patients with refractory ACP who had their ECMO reconfigured from conventional V-V or V-A to V-P ECMO. The clinical, biological, ventilatory, and hemodynamic characteristics of these patients are described in Supplementary Table S1. Patients’ age ranged between 20 and 57 years (3 women and 3 men). Five patients were initially supported by conventional V-V ECMO [for ARDS secondary to coronavirus disease of 2019 (COVID-19) pneumonia in three patients, acute chest syndrome in one, and pneumonia with asthma in one patient] and the 6th patient by V-A ECMO (for ARDS and refractory shock with RV failure due to acute chest syndrome). Cannulation was femoro-jugular for V-V ECMO and femoro-femoral for V-A ECMO. Patients were reconfigured to V-P ECMO after a median of 33 [1–67] days on conventional V-V or V-A ECMO. The latter was removed immediately after successful V-P ECMO implantation in five patients, and after one day in the first patient.

### Effect of reconfiguration

**Figure 3:**
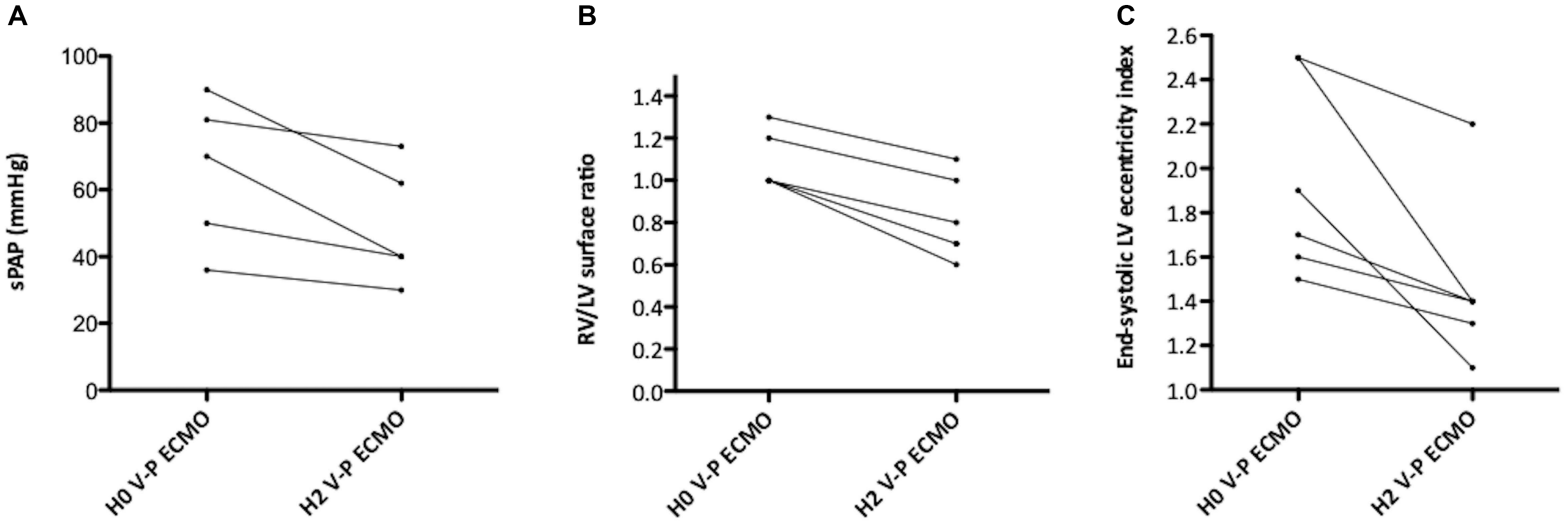
Right ventricle (RV) overload before and after conversion to veno-pulmonary arterial extracorporeal membrane oxygenation (V-P ECMO). **(A)** Change in systolic pulmonary artery pressure; **(B)** change in end-diastolic RV/left ventricle (LV) surface ratio; **(C)** change in end-systolic LV eccentricity index.

**Figure 4:**
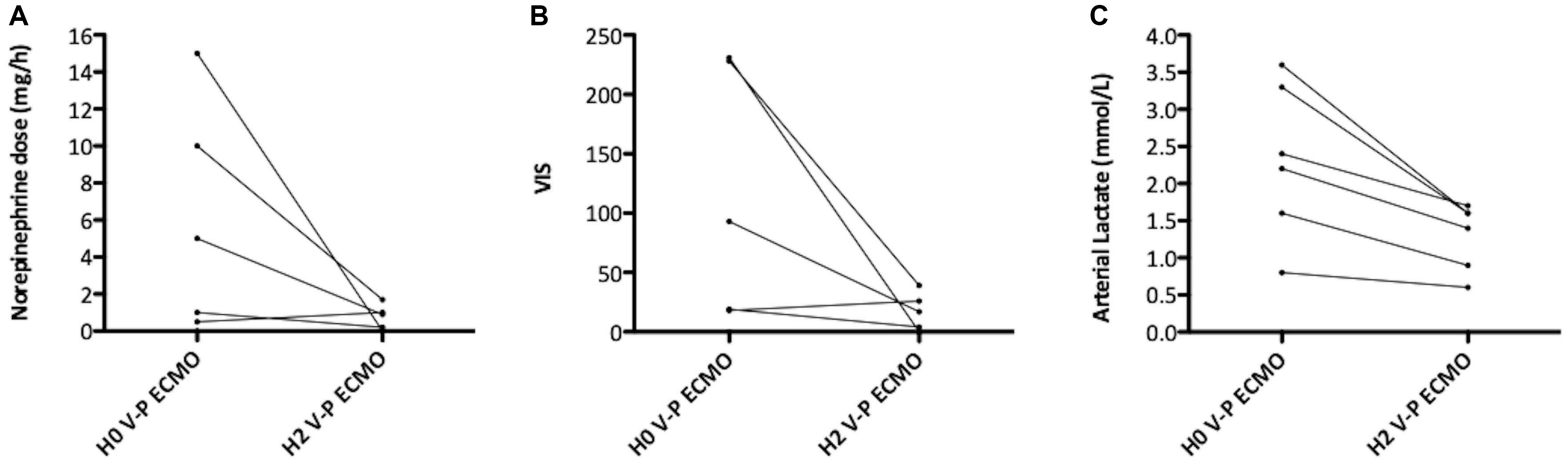
Hemodynamic variations upon reconfiguration to veno-pulmonary arterial extracorporeal membrane oxygenation (V-P ECMO). **(A)** Change in norepinephrine dose; **(B)** change in vasoactive inotropic score; **(C)** change in arterial lactate.

Echocardiographic, hemodynamic, and biological data of patients on conventional ECMO (before the reconfiguration) are presented in Supplementary Table S2. Once ECMO reconfigured, ACP improved in all patients, as evidenced by the decrease in sPAP, in RV/LV surface ratio and in end-systolic LV eccentricity index (Figure 3 and Supplementary material 3—video 2). Patients also showed a decrease in norepinephrine dose, VIS, and lactatemia, (Figure 4). Echocardiographic resolution of severe ACP was immediately documented in four (66%) patients. The RV systolic function (TAPSE) and coupling (RV-FAC/sPAP and TAPSE/sPAP) also numerically improved in most patients after V-P ECMO (Supplementary Table S2).

### Tolerance and outcomes

We did not observe any short-term procedural complication. We detected severe hemolysis in one patient on V-P ECMO requiring a high ECMO blood flow (4.5 L/min) to treat refractory hypoxemia. On the long term, two cases of right-sided infective endocarditis were suspected after prolonged V-P ECMO (41 and 11 days, respectively) in two patients presenting partial jugular thrombosis; their echocardiographic images were compatible with non-destructive vegetation on the tricuspid valve (Supplementary material 4—video 3), and several of their blood cultures were positive for coagulase-negative staphylococcus. However, the cultures of their cannulas remained negative. Patients were treated by prolonged antibiotic therapy without surgical management and showed favorable outcome. No secondary embolic events were detected. Another patient had suspected cannula-related thrombosis (without concomitant bacteremia), which disappeared after increasing the anticoagulant dose. After removal of ProtekDuo cannula, echocardiographic follow-up during the post-intensive care stay period did not reveal any lesion in the tricuspid valve, not even in the two suspected cases of right-sided infective endocarditis.

The V-P ECMO and mechanical ventilation were weaned after a median of 26 [8–93] and 95 [39–211] days after implantation, respectively. Five (83%) patients were discharged home and one patient did not recover and died in intensive care unit.

## Discussion

This report outlines the usefulness of V-P ECMO in patients with refractory ACP despite conventional V-V or V-A ECMO. The main findings of this case series are: (i) the rapid resolution of ACP once reconfigured to atrio-pulmonary assist bypassing the RV; (ii) the prolonged assistance with V-P ECMO may enable better rehabilitation, but with the potential risk of right-sided infective endocarditis.

Cannulation of the pulmonary artery has recently emerged as a promising method to improve extracorporeal membrane oxygenation management by enhancing RV function or reducing LV loading (23, 24). The ProtekDuo device is a single-site, dual-lumen (right atrium to pulmonary artery) cannula that was first introduced in 2016 as a percutaneous right ventricular assist device (RVAD) alongside the para-corporeal TandemHeart (CardiacAssist, Pittsburgh, PA) pump, particularly useful in case of postoperative RV failure following LVAD implantation (25–28). Then, an oxygenator was adjoined to the cannula, enabling the use of V-P ECMO in ARDS patients (29). Several studies have reported beneficial outcomes (such as improved survival and reduced renal failure) with cannula-mounted V-P ECMO, compared with conventional V-V ECMO, in patients with COVID-19-related ARDS, irrespective of RV function status (30–37). Specifically, the stability and the single-insertion site of the cannula are factors in favor of early extubation with awake ECMO, and consequently the early pulmonary rehabilitation, especially during prolonged support (29).

Our report differs from prior studies in several key points. First, the ProtekDuo was not the initial management device used in the study. Second, all our patients experienced severe RV failure with refractory ACP on conventional ECMO support. Our findings highlight the efficiency of V-P ECMO in alleviating refractory ACP by bypassing the RV. Historically, when RV failure persisted or worsened with V-V ECMO despite RV protective ventilation (15), conversion to V-A or V-A-V ECMO was considered the standard rescue strategy (17, 38). The theoretical advantage of V-P ECMO over the conventional one is its lower recirculation, which should potentially enhance oxygenation (39). Third, ARDS etiology was COVID-19 in half of our patients, and severe acute chest syndrome in two patients. COVID-19 and acute chest syndrome are forms of ARDS marked by significant pulmonary vascular dysfunction (40–42). The latter often has poor outcomes in patients put on conventional ECMO, hence the interest in V-P ECMO as a potentially superior alternative in such cases (43).

Moreover, a part from one patient, who had mild LV dysfunction at the time of V-P ECMO initiation, our patients maintained good LV systolic function. In contrast, high-blood flow V-P ECMO may lead to massive cardiogenic pulmonary edema in patients with LV systolic dysfunction, a complication not observed in our cohort.

Severe hemolysis happened in one patient due to the high V-P ECMO blood flow (4.5 L/min) required for adequate oxygenation. The risk of hemolysis can be reduced since the ProtekDuo cannula comes in two sizes (31 and 29 Fr), thus opting for the larger size in patients needing high blood flows (e.g., >4.5 L/min) is a preferable option (35). However, only the small size was available in our center during the study period. An innovative approach redesigning the ProtekDuo cannula as a double lumen return cannula supplemented by a femoral drainage cannula, has been described (35, 44) to offer higher blood flows (up to 7 liters per minute) and to improve oxygenation (35, 44). On the other hand, a high blood flow can also be achieved with a dual-site V-P ECMO strategy (35, 36, 39, 44). Other complications were reported on the ProtekDuo (28, 37), like intracannula thrombosis (45), right coronary artery compression (46), severe tricuspid regurgitation (47), and superior vena cava syndrome (48), yet none occurred in our patients. Altogether, our cohort did encounter two suspected cases of right-sided infective endocarditis in patients who had been on V-P ECMO mounted on ProtekDuo cannula for long time. While right-sided infective endocarditis related to conventional ECMO was not specifically documented (49), our two cases were associated with coagulase-negative *Staphylococcal* species, the most common organisms detected in ECMO-related bloodstream infections (49). Our findings are in accordance with a recent case series where two cases of right-sided infective endocarditis related to ProtekDuo cannula were reported (50). This potential specific complication of V-P ECMO warrants vigilant monitoring in future research.

Our report suffers from several limitations inherent to the study design. Being a retrospective study conducted in a single center on a very small sample, with no control group, makes the results only exploratory. With our data, we cannot draw conclusions on more patient-centered outcomes, such as mortality. Nonetheless, it should be noted that the decision to change the type of support for these patients is perilous and can only be envisaged in high volume ECMO centers with considerable expertise. Our preliminary data support the feasibility of initiating V-P ECMO in patients with refractory ACP on conventional ECMO. The precise indications of this technique warrant further research (39).

## Conclusion

We herein report the usefulness of V-P ECMO mounted on single-site, dual-lumen, right atrium-pulmonary artery cannula to treat refractory ACP in patients on conventional ECMO. V-P ECMO provided hemodynamic improvement and helped relief from refractory ACP. This support seems to allow a relatively long-term assist that may favor pulmonary rehabilitation, but the potential risk of right-sided infective endocarditis should be put under scrutiny in future studies.

## Data availability statement

The raw data supporting the conclusions of this article will be made available by the authors, without undue reservation.

## Ethics statement

The studies involving humans were approved by Mondor University Hospital’s ethics committee. The studies were conducted in accordance with the local legislation and institutional requirements. Written informed consent for participation was not required from the participants or the participants’ legal guardians/next of kin because this is an observational retrospective single-center study. In accordance with the ethical standards of our hospital’s institutional review board and French law, all patients or close relatives were informed that their personal data were collected in this research and that they could decline inclusion.

## Author contributions

FB: Conceptualization, Data curation, Formal analysis, Investigation, Methodology, Validation, Writing – original draft, Writing – review & editing. PM: Conceptualization, Data curation, Investigation, Writing – original draft. MB: Investigation, Writing – review & editing. CR: Investigation, Writing – review & editing. GS: Investigation, Writing – review & editing. AF: Investigation, Writing – review & editing. P-MC: Investigation, Writing – review & editing. ET: Investigation, Writing – review & editing. TF: Validation, Writing – review & editing. RG: Conceptualization, Investigation, Writing – review & editing. AM: Conceptualization, Methodology, Supervision, Validation, Writing – original draft.

## Glossary

ARDS: acute respiratory distress syndrome
ACP: acute cor pulmonale
RV: right ventricle
V-V ECMO: venovenous extracorporeal membrane oxygenation
V-A ECMO: veno-arterial extracorporeal membrane oxygenation
VAV ECMO: veno-arterio-venous extracorporeal membrane oxygenation
V-P ECMO: veno-pulmonary arterial extracorporeal membrane oxygenation
ICU: intensive care unit
LV: left ventricle
VIS: vasoactive inotropic score
sPAP: systolic pulmonary arterial pressure
FAC-RV: fractional area change of right ventricle
TAPSE: tricuspid annular plane systolic excursion
COVID-19: coronavirus disease of 2019
TEE: trans-esophageal echocardiography
RVAD: right ventricular assist device
