# Supplementary material for: Refractory cor pulmonale under extracorporeal membrane oxygenation for acute respiratory distress syndrome: the role of conversion to veno-pulmonary arterial assist—a case series
Source: Front Med (Lausanne). 2024 Apr 25;11:1348077. doi: 10.3389/fmed.2024.1348077 (PMC11079173; doi:10.3389/fmed.2024.1348077)
Supplement: Supplementary file 1 [file Table_1.docx]

| **TABLE S1** Characteristics and outcomes of patients reconfigured to veno-pulmonary arterial assist device for refractory acute cor pulmonale despite conventional extracorporeal membrane oxygenation. | | | | | | |
| --- | --- | --- | --- | --- | --- | --- |
| **Characteristics** | **Patient number** | | | | | |
|  | **1** | **2** | **3** | **4** | **5** | **6** |
| Age (y) | 23 | 37 | 43 | 34 | 57 | 20 |
| Sex | F | F | F | M | M | M |
| Body mass index (kg/m^2^) | 35.4 | 37.4 | 33.1 | 27.5 | 22.0 | 23.9 |
| Comorbidities | - | Sickle cell disease | Bariatric surgery | - | asthma | Sickle cell disease |
| SAPS-II at admission | 43 | 43 | 37 | 19 | 82 | 63 |
| Etiology of ARDS | COVID /D2 post-partum | Severe ACS | COVID | COVID | Severe asthma Bronchospasm | Severe ACS |
| ***Conventional ECMO*** |  | | | | | |
| Type of initial ECMO | V-V ECMO | V-V ECMO | V-V ECMO | V-V ECMO | V-V ECMO | V-A ECMO |
| PaO_2_/FiO_2_ at ECMO implantation | 60 | 49 | 60 | 62 | 223 | 200 |
| PaCO_2_ at ECMO implantation | - | - | - | - | 97 | 40 |
| Transfer on ECMO by mobile team from another hospital | Yes | Yes | Yes | Yes | No | No |
| MV duration before ECMO implantation (d) | 7 | 1 | 5 | 1 | 1 | 2 |
| Cannulation mode | Femoro-jugular | Femoro-jugular | Femoro-jugular | Femoro-jugular | Femoro-jugular | Femoro-femoral |
| Diameter of the admission /return cannula (Fr) | 29/21 | 27/21 | 27/21 | 27/21 | 25/21 | 23/19 |
| Number of prone position sessions (before/after ECMO) | 6/13 | 1/9 | 5/7 | 1/16 | 0/0 | 0/6 |
| Inhaled nitric oxide | Yes | Yes | No | No | Yes | Yes |
| SOFA | 8 | 13 | 8 | 4 | 13 | 15 |
| ***Ventilatory features**** |  | | | | | |
| Ventilation mode | BiPAP/APRV | BiPAP/APRV | BiPAP/APRV | BiPAP/APRV | BiPAP/APRV | ACV |
| FiO_2_ (%) | 60 | 60 | 100 | 70 | 100 | 40 |
| MO | 20 | 20 | 18 | 20 | 12 | 22 |
| Plateau pressure (cmH_2_0) | 24 | 24 | 24 | 26 | 28 | 19 |
| Total PEEP (cmH_2_0) | 12 | 12 | 10 | 12 | 14 | 8 |
| DP / Crs (cmH_2_0) | 12 / 10 | 12 / 6 | 14 / 7 | 14 / 9 | 14 / 13 | 11 / 29 |
| End tidal CO_2_ (mmHg) | 29 | - | 13 | 16 | 41 | 28 |
| Inhaled nitric oxide (ppm) | 10 | 10 | 20 | 10 | 10 | 10 |
| ***ECMO data**** |  | | | | | |
| ECMO blood flow (L/min) **†** | 5.0/2.0 | 4.6/3.8 | 5.4/3.5 | 6.6/3.6 | 4.7/3.7 | 4.1/2.4 |
| Sweep gas flow (L/min) **†** | 10/8 | 6/6 | 7/7 | 7/7 | 10/10 | 4/2 |
| Membrane oxygen fraction (%) | 100% | 100% | 100% | 100% | 100% | 50% |
| Duration of conventional ECMO (d) | 88 | 1 | 60 | 53 | 13 | 1 |
| Fluoroscopy time during ProtekDuo implantation (min) | - | 8 | 12 | 7 | 10 | 15 |
| ***Outcomes*** |  | | | | | |
| Tracheostomy | Yes | No | Yes | No | No | Yes |
| Invasive MV duration (d) | 207 | 28 | 224 | 146 | 44 | 42 |
| V-P ECMO duration (d) | 94 | 9 | 41 | 92 | 11 | 5 |
| Total ECMO duration (d) | 182 | 10 | 101 | 145 | 24 | 19 |
| Renal replacement therapy | No | Yes | No | Yes | Yes | Yes |
| ICU length of stay (d) | 201 | 37 | 249 | 146 | 58 | 45 |
| ICU mortality (d) | No | No | No | Yes | No | No |
| SAPS-II: Simplified Acute Physiology Score II; ACS: acute chest syndrome; ECMO: extracorporeal membrane oxygenation; PaO_2_: arterial oxygen tension; FiO_2_: fraction of inspired oxygen; PaCO_2_: arterial carbon dioxide tension; MV: mechanical ventilation; SOFA score: Sequential Organ Failure Assessment; BiPAP: bi-level positive airway pressure; APRV: airway pressure release ventilation; ACV: assist-control ventilation; PEEP: Positive end-expiratory pressure; DP: driving pressure; Crs: Respiratory system compliance; V-P ECMO: veno-pulmonary arterial extracorporeal membrane oxygenation; ICU: intensive care unit. *parameters just before Protek-Duo implantation; † before/after ProtekDuo. | | | | | | |

| **TABLE S2** Echocardiographic, hemodynamic, and biological data of patients reconfigured to veno-pulmonary arterial assist for refractory acute cor pulmonale after initially being on conventional extracorporeal membrane oxygenation. | | | | | | |
| --- | --- | --- | --- | --- | --- | --- |
| **Characteristics** | **Patient number** | | | | | |
|  | **1** | **2** | **3** | **4** | **5** | **6** |
| ***Echocardiographic data**** |  | | | | | |
| LVEF (%) | 70 | 75 | 55 | 50 | 60 | 45 |
| LV-GLS (%) | -15.2 | - | -10.7 | - | -19.4 | -8.4 |
| VTI LVOT (cm) **†** | 25/20 | - | 17/15 | 18 | 23/25 | 14/18 |
| CI (L/min/m^2^) **†** | 4.1/2.9 | - | 3.8/3.6 | 2.4/- | 3.2/2.8 | 3.3/3.9 |
| RV/LV surface ratio **†** | 1.0/0.6 | 1.2/1.0 | 1.0/0.7 | 1.0/0.8 | 1.3/1.1 | 1.0/0.7 |
| End-systolic LV ecc index **†** | 2.5/1.4 | 2.5/2.2 | 1.7/1.4 | 1.6/1.4 | 1.50/1.30 | 1.9/1.1 |
| TRV (m/s) **†** | 3.7/2.5 | 4.2/3.4 | 3.0/2.5 | - | 4.1/3.8 | 2.3/1.9 |
| sPAP (mmHg) **†** | 70/40 | 90/62 | 50/40 | - | 81/73 | 36/30 |
| FAC-RV (%) **†** | 28/37 | 20/30 | 18/20 | - | 21/36 | 22/34 |
| FAC/sPAP ratio**†** | 0.40/0.93 | 0.22/0.48 | 0.36/0.50 | - | 0.26/0.49 | 0.61/1.13 |
| Tric annular diam (mm) | 33 | 31 | 30 | - | 34 | 33 |
| TAPSE (mm) **†** | 16/19 | 10/14 | 18/15 | - | 14/16 | 16/21 |
| TASPE/sPAP **†** | 0.23/0.48 | 0.11/0.23 | 0.36/0.38 |  | 0.17/0.22 | 0.44/0.70 |
| S-tric (cm/s) **†** | 10/10 | 6/8 | 5/4 |  | 9/11 | 11/12 |
| RV-GLS (%) | -16.2 | - | -15.2 | - | -14.8 | -14.2 |
| RV-FWLS 3-segments avg (%) | -15.6 | - | -14.0 | - | -20.3 | -16.6 |
| RV-FWLS apical segments (%) | 5 | - | -5 | - | -25 | -11 |
| RV-FWLS medial segments (%) | -27 | - | - | - | - | -26 |
| RV-FWLS basal segments (%) | -15 | - | -25 | - | -15 | -15 |
| ***Hemodynamic data (D0 Protek-Duo)**** |  | | | | | |
| SBP / DBP (mmHg) | 103/58 | 109/47 | 88/60 | 99/50 | 96/59 | 108/49 |
| HR (bpm) **†** | 109/75 | 158/116 | 135/86 | 77/62 | 90/69 | 122/99 |
| CVP (mmHg) | 20 | 18 | - | - | - | - |
| Arterial Lactate (mmol/L) **†** | 2.4/1.7 | 3.3/1.6 | 1.6/0.9 | 0.8/0.6 | 3.6/1.8 | 2.2/1.4 |
| Norepinephrine dose (mg/h) **†** | 0/0 | 15/0 | 5.0/0.9 | 1.0/0.2 | 10.0/1.7 | 0.5/1.0 |
| Dobutamine (µg/Kg/min) **†** | 0/0 | 0/0 | 0/0 | 0/0 | 0/0 | 5/0 |
| VIS **†** | 0/0 | 116/0 | 46/8 | 10/2 | 114/19 | 12/13 |
| ***Arterial blood gas**** |  | | | | | |
| pH | 7.41 | 7.21 | 7.38 | 7.40 | 7.33 | 7.41 |
| PaO_2_ (mmHg)**†** | 67/240 | 84/136 | 53/380 | 74/80 | 51/154 | 132/113 |
| PaCO_2_ (mmHg) | 43/30 | 48/43 | 45/25 | 48/47 | 33/31 | 36/33 |
| Bicarbonates (mmol/L) | 28.3 | 20.0 | 28.1 | 30.5 | 19.0 | 23.3 |
| ***Hemolysis**** |  | | | | | |
| Serum free Hb (mg/L) **†** | 56/89 | 67/120 | 1980/39 | 277/172 | 135/627 | - |
| LDH (U/L) **†** | 752/630 | 1,248/3,527 | - | 509/472 | 1,011/1,483 | 2,312/1,457 |
| Bilirubin total/free (µmol/L) | 8.0/3.8 | 33.3/17.8 | 6.3/2.4 | 7.8/5.3 | 72.1/67.8 | 226.3/185.1 |
| HbCO (%) **†** | 3.2/2.8 | 2.1/1.6 | 2.3/2.6 | 3.5/3.1 | 1.2/1.2 | 5.3/4.6 |
| LVEF: left ventricular ejection fraction; LV-GLS: left ventricular global longitudinal strain; VTI LVOT: velocity–time integral of left ventricular outflow tract; CI: cardiac index; End-systolic LV ecc index: End-systolic Left ventricular eccentricity index; RV: right ventricle; LV; left ventricle; TRV: tricuspid regurgitation velocity; sPAP: systolic pulmonary arterial pressure; FAC-RV, fractional area change of right ventricle; Tric annular diam: tricuspid annular diameter; TAPSE: tricuspid annular plane systolic excursion; S-tric: peak of systolic tricuspid annulus velocity (obtained using pulsed tissue Doppler); RV-GLS: right ventricular global longitudinal strain; RVFWLS: RV free wall longitudinal strain; avg: average; SBP: systolic blood pressure; DBP: diastolic blood pressure; HR: heart rate; CVP: central venous pressure; VIS: Vasoactive Inotropic score (defined as: Dobutamine dose (μg/kg/min) + 100 x Epinephrine dose (μg/kg/min) + 100 x Norepinephrine dose (μg/kg/min); PaO_2_: partial arterial oxygen pressure; PaCO_2_: partial arterial carbon dioxide pressure; Hb: hemoglobin, LDH: lactate dehydrogenase; HbCO: carboxyhemoglobin. *parameters just before Protek-Duo implantation; † before/after Protek-Duo. | | | | | | |
